# Potential cost savings with terrestrial rabies control

**DOI:** 10.1186/1471-2458-7-47

**Published:** 2007-04-02

**Authors:** Sergio Recuenco, Bryan Cherry, Millicent Eidson

**Affiliations:** 1 Zoonoses Program, Bureau of Communicable Diseases Control, New York State Department of Health, 621 Corning Tower, Empire State Plaza, Albany, New York 12237, USA; 2 School of Public Health, University at Albany-SUNY, One University Place, Rensselaer, New York 12144, USA

## Abstract

**Background:**

The cost-benefit of raccoon rabies control strategies such as oral rabies vaccination (ORV) are under evaluation. As an initial quantification of the potential cost savings for a control program, the collection of selected rabies cost data was pilot tested for five counties in New York State (NYS) in a three-year period.

**Methods:**

Rabies costs reported to NYS from the study counties were computerized and linked to a human rabies exposure database. Consolidated costs by county and year were averaged and compared.

**Results:**

Reported rabies-associated costs for all rabies variants totalled $2.1 million, for human rabies postexposure prophylaxes (PEP) (90.9%), animal specimen preparation/shipment to laboratory (4.7%), and pet vaccination clinics (4.4%). The proportion that may be attributed to raccoon rabies control was 37% ($784,529). Average costs associated with the raccoon variant varied across counties from $440 to $1,885 per PEP, $14 to $44 per specimen, and $0.33 to $15 per pet vaccinated.

**Conclusion:**

Rabies costs vary widely by county in New York State, and were associated with human population size and methods used by counties to estimate costs. Rabies cost variability must be considered in developing estimates of possible ORV-related cost savings. Costs of PEPs and specimen preparation/shipments, as well as the costs of pet vaccination provided by this study may be valuable for development of more realistic scenarios in economic modelling of ORV costs versus benefits.

## Background

Rabies, caused by infection with a lyssavirus, has a worldwide distribution and has affected mankind since antiquity. Rabies is almost 100% fatal following serious neurological symptoms and great suffering [1]. Rabies has a significant impact on health system costs, both for animal rabies control and for human postexposure treatment [1]. Costs for rabies prevention have been estimated to be $230 million to $1 billion per year in the U.S. [2]. The recent increase in wildlife rabies, particularly in raccoons, has contributed significantly to the economic impact of rabies in the U.S., due to large increases in activities for prevention and control [3,4].

### U.S. rabies variants

The epidemiology of rabies in the U.S. has changed from a predominance in domestic animals to a predominance in wild animals [4]. Bat rabies variants are widely distributed, whereas terrestrial rabies variants are more focally distributed. Specific skunk variants are located in California, the northern Midwest, and the southern Midwest; fox variants are located in west Texas and southern Arizona; the dog/coyote variant is concentrated along the U.S./Mexico border; and the raccoon variant is located in eastern states [5]. Raccoon rabies, first identified in Florida in the 1940's, spread to the Mid-Atlantic States in the 1970's, and the northeastern U.S. in the 1990's [6,7]. In 1999, Canada reported its first epizootic of raccoon rabies [8].

In the eleven eastern states from North Carolina to New York (NY), 82% of the counties reported the presence of rabid raccoons during 1977–1997 [9]. Analyses of the geographic/temporal distribution of cases indicate that the raccoon rabies epizootic led to a subsequent epizootic of raccoon-variant rabies in skunks. Epizootics in both species moved in a similar direction from 1990 to 2000 [10]. Another study found that raccoon rabies spreads with a velocity of approximately 46 kilometers (km) per year [11]. The spread velocity of the raccoon epizootic for NY was estimated at 48 km per year northward [12]. Environmental barriers such as rivers slow the transmission as much as seven-fold from township to township, but do not stop it [11-13].

### Human postexposure treatment

A major cost associated with rabies is human postexposure prophylaxis (PEP), sometimes referred to as postexposure treatment. In the U.S., PEP consists of five doses of cell culture-derived rabies vaccine over the period of a month plus a single dose of rabies immune globulin [14]. PEP is expensive with a cost range of $1,038–$4,447 [15]. In NY, the average was $1,136 [16]. Studies suggest PEP is overused, which impacts overall costs [17,18]. Researchers estimated that PEP was unnecessary for about 40% of patients, while treatment had been inappropriately withheld in 6.3% of patients [19]. In areas currently free of raccoon rabies, PEP overuse is already a problem, and the spread of raccoon rabies will likely further increase PEP expenses [20]. When raccoon rabies reached Hunterdon and Warren counties in New Jersey, PEP cost was $1,100 per 100,000 population (pre-epizootic period), and by 1990 the cost was $74,734 per 100,000 population (epizootic period) [21].

### Animal vaccination

Primary prevention of rabies is based on animal vaccination [22], and many states require vaccination of pets [23]. An innovative wildlife rabies vaccination approach has used an oral rabies vaccine (ORV). ORV is used in 16 European countries, Israel, and North Africa for several animal species, including dogs, foxes, raccoon dogs, badgers, martens, wolves, and jackals [24-26]. In the U.S., a vaccinia-rabies glycoprotein recombinant vaccine (V-RG) is used. ORV is restricted to use in state or federally-approved rabies control programs and projects [22]. Its use is intended to reduce rabies-associated costs and prevent further spread of raccoon rabies to previously unaffected areas [23]. ORV has also been used for coyotes and foxes in Texas [24]. In Ontario, Canada the use of ORV between 1990 and 2000 reduced fox variant rabies cases by 90%. During the same period, a reduction of 50% in human PEPs was also documented [8].

Although the use of ORV in Canada and France has been linked with reductions in rabid animals [8,27], the administration of ORV programs is very expensive [28]. The benefits compared to costs can be high as in Europe in which highly concerted, nationally coordinated actions utilizing detailed knowledge of wildlife habitats and demography led to elimination of terrestrial rabies in Switzerland, Austria and large parts of Germany and France. To determine the cost/benefit ratio in the U.S., additional studies are required to document the economic impacts associated with raccoon variant rabies (which can be quantified as a 'benefit' of ORV programs in terms of costs avoided), determine the optimal bait density and the optimal distribution of the ORV baits, and better define the costs involved in an ORV program [28,29]. According to a recent study, preventing the westward spread of raccoon rabies in the U.S. is economically beneficial using ORV, even at an estimated cost between $58 million and $148 million for a 20-year control program [30].

### Rabies in NY

NY is representative of many states with multiple rabies variants and a mixture of urban, suburban and rural areas with a large human population potentially at risk for exposure [16,31]. Raccoon variant rabies was first detected in 1990 [12]. Initial studies indicate that raccoon rabies has had a significant impact on the epidemiology of rabies and associated health care costs. Raccoons represented 75% of the rabid animals from 1993–1998 [16]. Even prior to licensure of V-RG for use in raccoons, NY began a pilot ORV program in 1994. Initial cost estimates for a NY wildlife rabies control campaign including ORV were $10.7 million per year, and $73 million for a 10-year campaign [12]. The Department of Health (NYSDOH) baited two counties between the Adirondack Mountains and Lake Champlain to prevent northward rabies spread to Canada [31]. In 1996 Cornell University, in collaboration with the NYS Department of Environmental Conservation (NYSDEC) and the Ontario Ministry of Natural Resources, Canada, started an additional project to distribute baits in three northwestern counties [32]. In recent years, the United States Department of Agriculture's Wildlife Services has played a major role in both the NYSDOH and Cornell programs.

By law, NY places responsibility for rabies control and surveillance on the 57 local health departments (LHDs) outside New York City (NYC). NYSDOH operates a unique rabies reimbursement and reporting system for authorized PEPs (with third party medical insurance first applied to the cost of treatment), collection, preparation and submission of animal specimens to the State's Rabies Laboratory for testing, and five annual no-cost rabies vaccination clinics for dogs, cats and ferrets per county (recently reduced to three per year).

LHDs submit quarterly vouchers detailing expenses for these categories of rabies prevention activity. PEP expenses are reimbursed if the case has been reported using the NYSDOH reporting system, and are limited to a maximum of $1,000 per patient. For animal specimens, reimbursement requires reporting species, laboratory ID number, date of shipment, and cost of preparation/shipping, and is limited depending on species. Vaccination clinic reimbursement is limited to $5000 per county per year [33].

### Study objective

Previous studies of NY rabies PEP and rabies costs did not assess costs by variant [16,17] or variability by geographic location. To address these issues, this study used detailed voucher and reimbursement data and matched the records to the rabies exposure database. Reimbursement data was summarized by fiscal year rather than date of reimbursement.

To pilot test the computerization and analysis of rabies-control costs reported to NYSDOH, with emphasis on raccoon variant-associated costs to provide more specific cost estimates for ORV cost-benefit modelling, five counties were selected to represent diverse conditions of demographics, rabies epidemiology and reporting.

## Methods

### Data

This study includes reimbursement data from three consecutive fiscal years (April through March): 1999–2000, 2000–2001, and 2001–2002. Five counties were selected for the analysis, to represent diverse areas: the lower Hudson valley, upper Hudson valley, western NYS, central NYS, and northeastern NYS. One of the counties had an ORV program. Four counties have full service LHDs, including an environmental health program responsible for rabies exposure investigations. One of the LHDs is 'partial service' and relies on public health nurses for rabies follow-up. These counties represent 12.9% of the NY population, and 23.5% of the population outside NYC (Census 2000 data). With only one partial service county, which also was the only county with ORV, the association of cost data with being a partial service county and being exposed to ORV was not examined in this pilot study.

Vouchers were reviewed to identify PEPs begun during the study fiscal years, and vaccination clinics and specimen shipments during this same time period. A master list of names was used to link computerized databases. After data matching, cleaning, and extraction, names were deleted retaining identification codes from original data sources. Documents used for specimen preparation/shipment cost calculations were private carrier bills (UPS/Airborne Express), veterinarian bills/vouchers and information on personnel salaries.

Variables included expenses and dates for PEP treatments, specimen preparation/shipment, and vaccination clinics; amount paid by third party insurers for PEPs; amount of donations collected for vaccination clinics; amount reimbursed to LHDs by the State; and species involved in incidents or submitted for testing. When the amount of donations collected for vaccination clinics exceeded costs, the county cost was considered zero and the donation excess was not accounted in the total clinic cost.

LHD telephone interviews were used to determine how the LHDs calculated the specimen shipment costs and the information sources used for the calculations; and to understand the meaning of low values reported in the cost of PEPs and specimen shipments.

### Statistical analyses

To determine which control costs might be reduced or eliminated due to a successful ORV program, animal species involved in human rabies exposures were grouped into bats and non-bats (i.e., terrestrial). Most of the rodents were grouped together with the exception of squirrels and woodchucks because there were rabies cases in these species in recent years in the U.S. [34]. All terrestrial species whose frequencies were lower than ten animals were grouped as "other." Analyses were performed either excluding or including bats. Detailed analyses were conducted for costs related to terrestrial rabies.

The NYSDOH Wadsworth Center's Rabies Laboratory tests the variant for all rabid animals other than raccoons, and for a periodic sample of raccoons. A 20 year assessment identified variants other than raccoon (bat variants) for only 14 (0.1%) of 14,582 rabid terrestrial animals [35]. Thus, for this study, all exposures involving terrestrial animals were attributed to the raccoon variant.

In addition to a calculation of overall costs, statistics were calculated by county and fiscal year. Correlation coefficients were calculated for PEP and specimen costs with county population. A Pareto analysis was done for PEP and specimen costs by species.

To determine whether PEP costs due to terrestrial rabies are the same for patients exposed in the county of treatment and those exposed outside the treatment county, the average PEP cost was calculated and compared for the two groups. The null hypothesis was that the average cost of PEP was the same for both groups. This hypothesis was tested using Students' t-test for comparison of means. For this analysis we assumed the exposures are independent events.

Data management and statistical analyses were conducted using MS Access, MS Excel, and SAS. For graphics S-Plus, MS Excel and MS PowerPoint were used.

## Results

### Overall costs

For the study counties, the total cost of prevention activities for both terrestrial and bat rabies during fiscal years 1999–2001 was $2,143,905. Of this total, $1,948,008 (90.9%) represented PEP costs, $100,278 (4.7%) represented specimen preparation/shipment costs, and $95,618 (4.4%) represented pet vaccination clinic costs. Table 1 presents the proportion of these expenses related to raccoon rabies variant, by individual study county and payer. Because vaccination clinic costs may be incurred regardless of rabies variant, they are included in Table 1 but cannot be definitively attributed to the presence of raccoon variant rabies.

**Table 1:** Raccoon-variant rabies costs, fiscal years 1999–2000 to 2001–2002, in five selected counties of NYS (with percentage total cost across all variants attributable to raccoon rabies)

		**County A**		**County B**		**County C**		**County D**		**County E**		**Total**	
							
		**$**	**%**	**$**	**%**	**$**	**%**	**$**	**%**	**$**	**%**	**$**	**%**
***PEPs***	***Total***	$89,801.05	39.99%	$7,861.71	12.15%	$58,547.63	48.39%	$147,027.28	54.57%	$315,036.04	24.84%	$618,273.71	31.74%
	***State***	$53,303.38	41.65%	$723.21	6.07%	$23,723.80	47.12%	$36,590.63	69.35%	$193,128.73	27.84%	$307,469.75	32.83%
	***County***	$5,735.18	32.99%	$0.00	0.00%	$7,327.49	59.32%	$12,692.44	73.54%	$21,808.87	32.83%	$47,563.98	40.93%
	***Third party***	$30,762.49	38.85%	$7,138.50	14.28%	$27,496.34	47.16%	$97,744.21	49.01%	$100,098.44	19.70%	$263,239.98	29.41%
													
***Specimens***	***Total***	$74.00	100.00%	$1,631.95	75.94%	$10,401.16	60.40%	$10,734.02	62.80%	$47,796.31	74.98%	$70,637.44	70.44%
	***State***	$74.00	100.00%	$1,579.25	75.61%	$9,569.26	58.48%	$10,734.02	62.80%	$42,639.89	74.00%	$64,596.42	69.28%
	***County***	$0.00	0.00%	$52.70	87.22%	$831.90	97.05%	$0.00	0.00%	$5,156.42	84.20%	$6,041.02	85.79%
													
***Vaccination***	***Total***	$25,946.24	(N.A.)	$20,470.83	(N.A.)	$3,237.50	(N.A.)	$44,763.60	(N.A.)	$1,200.00	(N.A.)	$95,618.17	(N.A.)
***Clinics***	***State***	$6,501.74	(N.A.)	$9,832.54	(N.A.)	$3,213.75	(N.A.)	$13,614.08	(N.A.)	$1,200.00	(N.A.)	$34,362.11	(N.A.)
	***County***	$0.00	(N.A.)	$0.00	(N.A.)	$23.75	(N.A.)	$589.78	(N.A.)	$0.00	(N.A.)	$613.53	(N.A.)
	Donations*	$19,685.00	(N.A.)	$10,638.29	(N.A.)	$0.00	(N.A.)	$32,435.29	(N.A.)	$0.00	(N.A.)	$62,758.58	(N.A.)

*Donations for vaccination clinics. The excess in donations reported in counties A and D were not accounted for in the vaccination total cost. N.A.: Proportion attributable to raccoon variant is not applicable to vaccination clinic costs.

The total expenses that may be attributed to terrestrial rabies in this study were $784,529 (36.6%) for the three-year period, if including all vaccination clinic costs. Without clinic costs, the total was $688,911 (32.1%) (Table 1). By fiscal year the terrestrial rabies-associated expenses (including clinic costs) were $247,700 in 1999–2000, $261,805 in 2000–2001, and $277,140 in 2001–2002 (Table 2). The NYSDOH reimbursement system provided funds to cover 52% of the total terrestrial rabies-associated costs ($406,428), about 34% was covered by third party health insurers (for PEPs), about 8% was covered by donations for vaccination clinics, and about 7% was covered by the LHDs.

**Table 2:** Terrestrial rabies costs by fiscal year and county

**County**	**1999–2000**	**2000–2001**	**2001–2002**	**Total**
***Postexposure Prophylaxis***				
				
A	$25,782.27	$48,655.38	$15,363.40	$89,801.05
B	$2,122.21	$2,103.25	$3,636.25	$7,861.71
C	$20,828.65	$13,441.87	$24,277.11	$58,547.63
D	$59,738.40	$62,359.17	$24,929.71	$147,027.28
E	$81,887.23	$81,931.73	$151,217.08	$315,036.04
All counties	$190,358.76	$208,491.40	$219,423.55	$618,273.71
				
***Specimen Submission***				
				
A	$0.00	$74.00	$0.00	$74.00
B	$411.00	$666.50	$554.45	$1,631.95
C	$6,250.16	$1,516.96	$2,634.04	$10,401.16
D	$4,310.68	$3,307.70	$3,115.64	$10,734.02
E	$13,592.06	$15,450.43	$18,753.82	$47,796.31
All counties	$24,563.90	$21,015.59	$25,057.95	$70,637.44
				
***Vaccination Clinics***				
				
A	$8,778.75	$9,552.84	$7,614.65	$25,946.24
B	$6,101.45	$6,924.33	$7,445.05	$20,470.83
C	$1,023.75	$1,153.75	$1,060.00	$3,237.50
D	$15,046.67	$13,807.77	$15,909.16	$44,763.60
E	$0.00	$570.00	$630.00	$1,200.00
All counties	$30,950.62	$32,008.69	$32,658.86	$95,618.17

The average per capita cost for terrestrial rabies including PEPs, specimen preparation/shipment, and pet vaccination clinics was $0.32, with a range of $0.10 to $0.77.

### PEP costs

PEP was administered to 2,085 persons in the five counties during the 1999–2002 fiscal years. Terrestrial animal exposures were associated with 657 PEPs (32%). Terrestrial rabies-associated PEP costs were $618,274 (32%) (Table 1). Costs vary by fiscal year (Table 2), increasing from $190,359 in 1999–2000 to $219,424 in 2001–2002. The State reimbursed $307,470 (49.7%) of the terrestrial rabies-associated costs, $263,240 (42.6%) was covered by third party payers, and $47,564 (7.7%) was paid by LHDs (Table 3). The average terrestrial rabies-associated PEP cost was $941.06, with a range of $440.21 to $1,884.97 per county (Table 3).

**Table 3:** Terrestrial rabies costs by payer and county, NYS fiscal years 1999–2000 to 2001–2002

**County**	**Number of persons treated, specimens submitted, or animals vaccinated**	**State Cost**	**Third Party Cost**	**Donations**	**County Cost**	Average Cost*
***Post exposure prophylaxis***						
						
A	126	$53,303.38	$30,762.49	-	$5,735.18	$712.71
B	7	$723.21	$7,138.50	-	$0.00	$1,123.10
C	133	$23,723.80	$27,496.34	-	$7,327.49	$440.21
D	78	$36,590.63	$97,744.21	-	$12,692.44	$1,884.97
E	313	$193,128.73	$100,098.44	-	$21,808.87	$1,006.50
All counties	657	$307,469.75	$263,239.98	-	$47,563.98	$941.06
						
***Specimen Submission***						
						
A	2	$74.00	-	-	$0.00	$37.00
B	114	$1,579.25	-	-	$52.70	$14.32
C	321	$9,569.26	-	-	$831.90	$32.40
D	503	$10,734.02	-	-	$0.00	$21.34
E	1078	$42,639.89	-	-	$5,156.42	$44.34
All counties	2,018	$64,596.42	-	-	$6,041.02	$35.00
						
***Vaccine Clinics***						
						
A	4140	$6,501.74	-	$19,685.00	$0.00	$6.27
B	3309	$9,832.54	-	$10,638.29	$0.00	$6.19
C	9915	$3,213.75	-	$0.00	$23.75	$0.33
D	8741	$13,614.08	-	$32,435.29	$589.78	$5.12
E	80	$1,200.00	-	$0.00	$0.00	$15.00
All counties	26,185	$34,362.11	-	$62,758.58	$613.53	$3.65
						
***All Categories***						
						
A	-	$59,879.12	$30,762.49	$19,685.00	$5,735.18	-
B	-	$12,135.00	$7,138.50	$10,638.29	$52.70	-
C	-	$36,506.81	$27,496.34	$0.00	$8,183.14	-
D	-	$60,938.73	$97,744.21	$32,435.29	$13,282.22	-
E	-	$236,968.62	$100,098.44	$0.00	$26,965.29	-
All counties	-	$406,428.28	$263,239.98	$62,758.58	$54,218.53	-

* Average cost = (cost/n)

**Figure 1:**
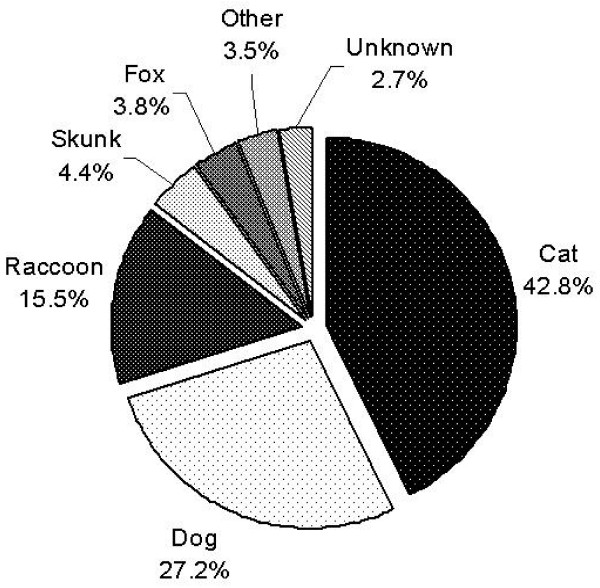
Terrestrial species associated with PEPs, five counties of NYS, fiscal years 1999–2000 to 2001–2002.

Cats were associated with 43% (281) of the terrestrial rabies PEPs, dogs with 27% (179), and raccoons with 16% (102) (Figure 1). Other species were reported for 14% of the PEPs, including skunk, fox, woodchuck, cattle, ferret, coyote, beaver, squirrel, monkey, opossum, porcupine, rabbit, tiger, and wolf. PEP costs associated with dogs and cats were $437,225, 70.7% of the total terrestrial rabies-associated PEP costs. The only county with an ORV program had no PEP due to raccoon exposures. A Pareto analysis of PEP costs by species indicates that about 86% are due to incidents involving cats, dogs and raccoons, and 70% of the PEPs were due to contacts with pets. During the study period, only three dogs were confirmed with rabies and none in the study counties, compared to a total of 18 cats (all confirmed to have raccoon variant).

Forty-three (6.5%) of the patients receiving PEP due to terrestrial rabies exposures were exposed in a county other than the one providing the treatment and twenty-nine (4.4%) were exposed outside the State or country. The average PEP cost for patients exposed in the county providing the treatment was $959.25, compared to an average cost for patients exposed elsewhere of $793.19 (t = 2.08, df = 655, p = 0.0383). The association of this factor (treatment within or outside exposure county) with completion of treatment was not assessed.

The average per capita cost for terrestrial rabies PEPs during the three-year study period was $0.25, with a range of $0.08 to $0.34. The county population size was highly correlated with the number of PEPs (r = 0.86) and PEP costs (r = 0.75), but not with PEP average cost (r = -0.21). A cost < $300 was reported for 132 terrestrial rabies-associated PEPs. LHDs reported these represented patients not receiving a full five-dose vaccine series and immune globulin, PEP for lower weight children (because HRIG costs are based on body weight), or incomplete submission of bills. The reasons provided for not receiving a full treatment series included repeat PEPs that only require two doses of vaccine, stopping the PEP when the animal was found not to be rabid, and stopping the PEP after it was started by an emergency room, private doctor or health center out of county after determining it was not needed. Having the exposure and PEP in different counties was also reported as a cause for incomplete submission of bills to the LHD.

### Specimen costs

During the study period, 4,393 specimens were submitted for rabies testing from the study counties at a cost of $95,618. About 46% (2,018) of the specimens were terrestrial animals, but these specimens accounted for 70% of the submission costs ($70,637) (Table 1). The State reimbursed 91.4% ($64,596) of the terrestrial animal specimen submission costs.

The average cost of terrestrial animal specimen preparation/shipment was $35, with variation across counties (Table 3). The total cost for 14 large animal (horse, cattle) specimens was $278.34, at an average cost of $19.88. For all other specimens, the total cost was $70,359.10, at an average cost of $35. It is unexpected to find lower reported costs for large animal specimens because LHDs are permitted to voucher a larger amount for them. Because horses and cattle are found normally on farms, preparation of the specimens may be assumed as part of the regular veterinary work and not reported as associated with rabies testing.

**Figure 2:**
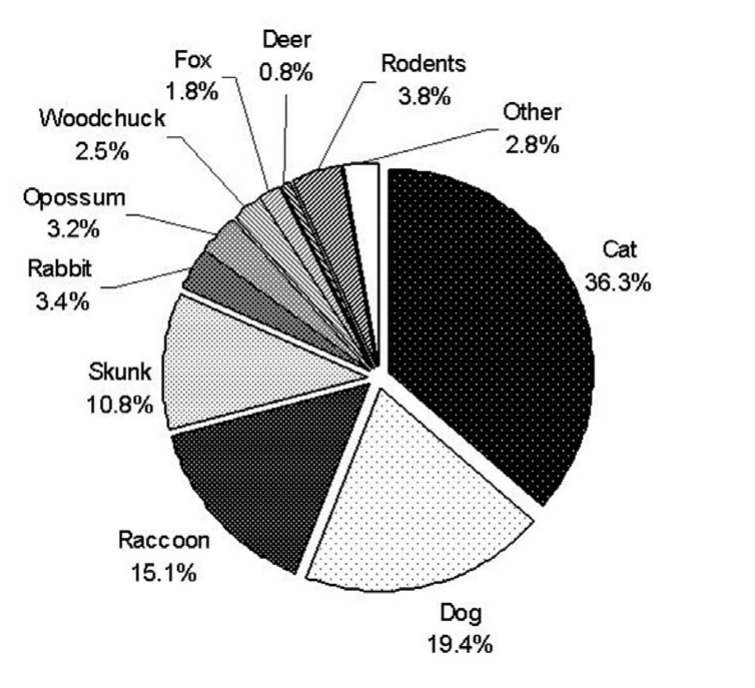
Species submitted for laboratory testing associated with terrestrial rabies incidents, five counties of NYS, fiscal years 1999–2000 to 2001–2002.

Regarding species submitted for testing, cats comprised 36% (733); dogs 19% (392); raccoons 15% (305), and skunks 11% (218) (Figure 2). Rodents, rabbits, opossum, woodchuck, fox, and deer comprised < 4% each, with other species making up < 3%. A Pareto analysis indicates that about 82% of the terrestrial specimen expenditures were for cats, raccoons, skunks, and dogs.

The average per capita cost for terrestrial rabies specimen preparation/submission was $0.03 (range = $0.0003–$0.05). The county population size was highly correlated with the number of specimens (r = 0.80), specimen preparation/shipment costs (r = 0.81), and the average specimen cost (r = 0.76).

LHD interviews indicated that veterinarians are contracted for specimen preparation in three counties. One county uses its own personnel. In two counties, veterinarians are paid according to the species, whereas in a third county veterinarians are paid a fixed fee per specimen. However, the reimbursement vouchers for that county did not itemize the veterinarian fee and the vouchers reported varying costs. In one county, veterinarians ship the specimens and report the cost to the LHD. Two counties report no cost for some shipments, usually for bats. Although shipment costs vary based on specimen weight, one county calculates a fixed-cost per specimen for reimbursement purposes. The formula is based on the average time to prepare a specimen, staff salary for that time, and average shipping costs.

### Vaccination clinic costs

There were 26,185 animals vaccinated in study county-sponsored clinics. The total cost was $95,618, of which $34,362 (36%) was reimbursed by NYSDOH (Table 1), $62,758 (66%) was from owner donations, and $614 (1%) was covered by LHDs. Little change was observed in the annual clinic costs through the study period (Table 2). Vaccination costs varied by county, from $1200 to $44,764 (Table 2).

As reported by four counties, 57% of the vaccinated animals were dogs and 43% were cats. The average cost per animal was $3.65, with variation from $0.33–$15.00 by county. Lower values under vaccine purchase prices likely indicate incomplete data. The average per capita cost for pet vaccination clinics was $0.04 (range = $0.004–$0.53). County population size was not correlated with the number of animals vaccinated (r = -0.02), but was inversely correlated with clinic total costs (r = -0.57) and partially correlated with the average cost per animal vaccinated (r = 0.32).

## Discussion

The cost summary presented in this pilot study is an initial quantification of rabies reimbursement data in NYS for PEPs, specimen preparation/shipment, and pet vaccination for five counties over a three-year time period. This data may assist in determining the parameters for the potential savings to be achieved if ORV or some other prevention/control program is used to reduce or eliminate the raccoon rabies virus variant in an eastern U.S. state. This study found that terrestrial rabies accounts for more than a third (37%) of the reimbursed rabies prevention and control costs. The study found that in NYS, the State bears the largest proportion of the reported costs. About half of all expenses were covered by the State in the five counties studied. The LHDs covered 7% of the total expenses. While the amount spent by LHDs is a small proportion of the total costs of terrestrial rabies control reported in this study, for some counties this may represent an important impact to their budget.

The per-capita cost of terrestrial rabies including PEP, specimen cost and pet vaccination clinics costs was estimated to be $0.32. However, use of this average in estimates of potential savings from ORV may considerably over- or under-estimate the potential savings as indicated by the county variation in average costs from $0.10–$0.77 per person. Although counties with larger population sizes had greater PEP and specimen costs, the per capita cost was highest in the smallest county, perhaps reflecting economies of scale in larger counties able to prorate their costs over a larger number of exposures. The fact that total terrestrial variant-reimbursed costs are increasing despite a relatively stable enzootic state of rabies in NYS, and active prevention and control programs, should be considered when estimating the future savings potentially associated with use of control programs.

### PEP

PEP expenses account for about 90% of the overall costs reported in this study. Thus, PEP is a large component of possible cost savings to be produced by public health intervention to control raccoon rabies. If ORV is used effectively to eliminate the raccoon variant, raccoon attacks are likely to be minimized and there will be no spillover of rabies to other species from this variant, thus reducing the total number of rabid animals and human exposures. In this study about 16% of the PEPs attributed to raccoon variant rabies were related directly to raccoon incidents, and 4% were due to skunk incidents. With raccoons and skunks unlikely to attack people if not rabid, it can be assumed that raccoon rabies elimination would eliminate most of the PEPs related to these species. Exposure to these two species represented 131 incidents for the 5 counties in the 3-year study period, at an estimated PEP cost of $125,271 representing 16% of the total terrestrial-associated costs.

Humans have more frequent contact with domesticated species than with wildlife, and bites to humans from these species frequently occur for reasons unrelated to rabies. In this study bites from dogs and cats accounted for 70% of the total terrestrial-associated PEP costs. Although elimination of the raccoon variant with ORV would reduce the number of rabid pets and other domestic animals and thus exposures to rabies, bites from pets will continue. Thus elimination of raccoon variant would result in a reduction in PEP costs associated with dog and cat incidents, but the reduction may not be substantial because most dog and cat bites are not due to the animal being rabid. PEP costs associated with pet exposures will likely not decline significantly until there is sufficient assurance that the raccoon variant has truly been eliminated and the probability of a pet being rabid is sufficiently low that PEP is not warranted even when the pet's rabies status cannot be verified. Changes in policies about providing PEP when a pet's rabies status cannot be determined would require a sufficient period without raccoon variant and without it encroaching from neighboring areas. These changes also assume no other terrestrial variants with frequent spillover to pets. If the raccoon variant had been eliminated from these five counties during the three-year study period and there was sufficient confidence that terrestrial animal-related exposures did not require PEP consideration, the maximum potential savings would have been 657 PEPs avoided at an estimated cost savings of $618,274.

### Animal specimens

With control of raccoon variant rabies, there should be fewer wildlife specimens to submit. This study found that 70% of the specimens submitted for testing were for terrestrial species, primarily dogs, cats, raccoons and skunks. If all raccoon and skunk submissions were eliminated by eliminating raccoon variant rabies, the cost savings for the study period would have been 26% of the total cost for terrestrial rabies specimen preparation/shipment, representing a savings of 523 specimens that would not have been prepared and shipped. This would yield an estimated cost savings of $24,228, accounting for 3% of the total terrestrial-associated costs.

Dog and cat specimens are submitted when the animal is showing signs of rabies because neurologic animals must be euthanized and tested after a bite to a human. Even with elimination of the raccoon variant, there will be occasional bites from neurologic pets due to bat rabies or other diseases. However, specimen shipments are likely to be considerably reduced. In NYS, avoiding 4,393 specimen shipments for all species at a cost savings of $95,618 is an upper limit of the potential savings in five counties over a three-year period.

### Vaccination clinics

NYS law requires rabies vaccination of pet dogs, cats and ferrets. This requirement would not be modified in a scenario where ORV controlled or eliminated raccoon rabies because bat variants remain. The possibility of translocation or reintroduction of rabies in wildlife also remains, therefore pets will continue being vaccinated.

An elimination of terrestrial rabies could lead to a reconsideration of the need for State resources to pay for pet vaccination clinics. Thus, State-associated costs of $34,362 could be considered a possible savings to be achieved with ORV. However, if pet vaccination continues as recommended by national guidelines and required by state laws, total vaccination costs may potentially increase if the costs associated with individual veterinarian vaccinations are higher than mass clinic costs. The current study indicates a wide disparity in the per-animal cost of rabies vaccination, ranging from $0.33 for a large county that offers mass vaccination clinics to $15.00 for a large county that reimburses a flat fee to veterinarians for vaccinating pets at their regular practice.

## Conclusion

An initial pilot study of the reimbursements provided for rabies costs in five NYS counties over a three-year period indicates a wide variability in costs by county. Counties with larger populations experienced larger PEP and specimen shipment/preparation costs. The study indicated that there are wide variations in the ways that LHDs estimate rabies-associated costs. Such potential variation must be considered in developing estimates of possible ORV-related cost savings. Within the limitations of LHD estimations of cost data, this study offers specific cost data for two critical factors, PEPs and specimen preparation/shipments, as well as the costs of pet vaccination. Actual data such as that obtained in this pilot study are critical for basing the parameters chosen for economic modelling of potential ORV benefit on realistic scenarios.

### Limitations

Interviews with counties indicated that cost data is not universally complete or accurate if based on estimates. Year-specific costs may be affected by lags in reporting PEP costs associated with insurance paperwork.

Overall averages for specimen preparation/shipment were artificially lowered by the inclusion of a county with the NYSDOH Rabies Laboratory which can hand deliver specimens and thus incurs almost no specimen shipment costs. The unexpected lower average cost for large animal specimen preparation/shipment may also lead to an underestimate of the average cost for all specimens. In one county, species vaccinated was not reported, and in another county, the reported cost per animal was below the actual cost of vaccine and other supplies.

The five upstate New York counties may not be representative of other areas, although they represent almost a quarter of the State's non-NYC population. With a larger study, factors such as population density, health care access, LHD capabilities, and animal rabies incidence could be assessed for their impact on rabies-associated costs. The influence of ORV itself could not be estimated with only one ORV county in the study.

Assuming that these costs associated with raccoon rabies would all be saved if ORV eliminated the raccoon variant is only applicable to areas with no other terrestrial variants. With bats having an insignificant impact on rabies in most other species (except humans), eliminating the raccoon variant could indeed result in elimination of most of the costs associated with the current raccoon-variant outbreak. However, PEP costs are clearly the largest component of the costs documented in this study. Until health care and public health professionals are sufficiently confident of the low risk of rabies from terrestrial animal exposures in areas where terrestrial variants have been eliminated, PEPs will continue to occur in relation to these exposures. With the evidence of raccoon variant in skunks tracking similarly to the raccoon variant in raccoons [10], estimating that all of the costs associated with raccoon variant could be eliminated with ORV for raccoons may also be inaccurate if skunks are now serving as an alternative reservoir for this variant. Skunks do not readily take the raccoon ORV bait nor are they as responsive to immunization with V-RG.

The most significant limitation on potential PEP savings from terrestrial variant control is the fact that in NYS only 31% of the PEPs were due to terrestrial animal exposures, representing only 29% of the total PEP costs. There are also many other rabies-associated costs that require better data in order to appropriately model potential ORV-associated savings, including costs associated with animal confinements, quarantines, and euthanasias, personal costs to those receiving PEP including time lost, pain and suffering, and costs to governmental agencies for managing the rabies cases and the consequent consulting, investigation, and prevention/control measures.

### Future research

The current study has demonstrated the feasibility of obtaining actual cost data for several important rabies-associated cost variables, and the variability in such costs across time and geographic area. Additional studies are required to include a larger area and additional important cost variables. In the interim, the data from this pilot study may be helpful in refining the parameters used in any additional ORV cost-benefit analyses for the elimination or reduction of raccoon-variant rabies, including modelling studies of animal to human transmission risks. In these studies, it will be critical to include varying levels of bait density and geographic size of baited areas, in addition to estimates of vaccine efficacy, as components of the cost-benefit assessments.

## Competing interests

The author(s) declare that they have no competing interests.

## Authors' contributions

SR carried out the computerization of the cost data, performed statistical analysis and drafted the manuscript. BC participated in the analysis design and result interpretation. ME conceived of the study, and participated in its design and coordination and helped to draft the manuscript. All authors read and approved the final manuscript.

## Pre-publication history

The pre-publication history for this paper can be accessed here:


